# Perception and Production of Statement-Question Intonation in Autism Spectrum Disorder: A Developmental Investigation

**DOI:** 10.1007/s10803-021-05220-4

**Published:** 2021-08-05

**Authors:** Li Wang, C. Philip Beaman, Cunmei Jiang, Fang Liu

**Affiliations:** 1 School of Psychology and Clinical Language Sciences, University of Reading, Reading, UK; 2 Music College, Shanghai Normal University, Shanghai, China

**Keywords:** Autism spectrum disorder, Speech, Music, Intonation, Pitch

## Abstract

Prosody or “melody in speech” in autism spectrum disorder (ASD) is often perceived as atypical. This study examined perception and production of statements and questions in 84 children, adolescents and adults with and without ASD, as well as participants’ pitch direction discrimination thresholds. The results suggested that the abilities to discriminate (in both speech and music conditions), identify, and imitate statement-question intonation were intact in individuals with ASD across age cohorts. Sensitivity to pitch direction predicted performance on intonation processing in both groups, who also exhibited similar developmental changes. These findings provide evidence for shared mechanisms in pitch processing between speech and music, as well as associations between low- and high-level pitch processing and between perception and production of pitch.

**Supplementary Information:**

The online version contains supplementary material available at 10.1007/s10803-021-05220-4.

## Introduction

Prosody is a suprasegmental feature of speech that adds additional pragmatic, affective, or grammatical information via changes in frequency, intensity, and duration of spoken utterances (McAlpine et al., 2014; McCann & Peppé, 2003; Paul et al., 2005). It plays an important role in speech communication and social interaction (Xu, 2005). While the acquisition of prosody starts from infancy (Levitt, 1993) and lays the foundations for children’s sociopragmatic development (Hübscher & Prieto, 2019), atypical prosody can become a barrier to everyday linguistic and social functioning, as seen in autism spectrum disorder (ASD) (Lloyd-Fox et al., 2013; McCann & Peppé, 2003; Paul et al., 2005).

ASD is a neurodevelopmental disorder associated with deficits in social communication and interaction as well as restricted and repetitive behaviours and interests (American Psychiatric Association, 2013). Prosodic deficits have been frequently observed in ASD across a variety of perception and production tasks (Diehl & Paul, 2012; Nakai et al., 2014; Peppé et al., 2007; Shriberg et al., 2011; Tager‐Flusberg et al., 2005). They can occur even in highly verbal individuals with ASD and tend to be lifelong even when other areas of language, such as semantics and syntax, improve (McCann & Peppé, 2003). Among the different areas of prosody, recognising and differentiating the rising from falling intonation in questions and statements represents an important aspect of conversational and linguistic competence (Dahan, 2015; Xie et al., 2021), and the literature in ASD has produced mixed findings (Chevallier et al., 2009; Filipe et al., 2014; Järvinen-Pasley et al., 2008a; Jiang et al., 2015; McCann & Peppé, 2003; McCann et al., 2007; Paul et al., 2005; Peppé et al., 2007). The current study investigated this issue by examining the roles of response bias, stimulus type, age, and pitch discrimination thresholds in the perception and production of statement-question intonation in ASD.

### Perception of Statement-Question Intonation and Response Bias in ASD

Several studies used the same test battery, PEPS-C (Profiling Elements of Prosodic Systems-Children) (Peppé & McCann, 2003), to examine discrimination (e.g. same vs. different) and identification (e.g. question vs. statement) of statements and questions in ASD (Filipe et al., 2014; Järvinen-Pasley et al., 2008a; McCann et al., 2007; Peppé et al., 2007). Within this battery, statement-question identification is assessed using a turn-end task with single words, e.g. “Carrots.” vs. “Carrots?”. Statement-question discrimination is assessed within a short-item discrimination task, which contains the laryngographic sounds (devoid of meaning) of the statement-question pairs, as well as those of the liking-disliking pairs, e.g. “tea” pronounced as though the speakers like it or dislike it, from the affect subtask in PEPS-C. Thus, the identification and discrimination tasks are unmatched in stimulus type (speech vs. laryngographic sounds) and in the number of relevant stimuli (only statements or questions are used in the identification task, whereas both statement-question and liking-disliking pairs are included in the discrimination task) in these studies (Filipe et al., 2014; Järvinen-Pasley et al., 2008a; McCann et al., 2007; Peppé et al., 2007). Results from these studies suggest that individuals with ASD are unimpaired in statement-question identification compared to typically developing (TD) peers (Filipe et al., 2014; Järvinen-Pasley et al., 2008a; McCann et al., 2007; Peppé et al., 2007). However, impaired discrimination between statements and questions was observed in one sample of participants (31 ASD vs. 72 TD participants) (McCann et al., 2007; Peppé et al., 2007), while a different sample showed intact discrimination (21 ASD vs. 21 TD participants) (Järvinen-Pasley et al., 2008a). In summary, studies using PEPS-C suggest intact statement-question identification but the results on statement-question discrimination in ASD are unclear, in part due to limitations of the design, as well as mixed results from different studies (Filipe et al., 2014; Järvinen-Pasley et al., 2008a; McCann et al., 2007; Peppé et al., 2007).

Using prosodic tasks other than PEPS-C (e.g. sentence stimuli from Patel et al., 1998), several studies also reported intact statement-question identification beyond single-word stimuli in ASD (Chevallier et al., 2009; Järvinen-Pasley et al., 2008a; Paul et al., 2005). However, using disyllabic phrases from Jiang et al. (2010), Jiang et al. (2015) revealed impaired identification and discrimination of statement-question intonation in Mandarin speakers with ASD. While the different results between Jiang et al. (2015) and other studies (Chevallier et al., 2009; Järvinen-Pasley et al., 2008a; Paul et al., 2005) may be attributed to the different language or cultural background of the participants: Mandarin Chinese versus English, it may also be the case that the discrepancy was due to differences in task difficulty across these studies. Indeed, participants from both the ASD and TD groups performed at ceiling in Paul et al. (2005), which used the stimuli from Patel et al. (1998). In that stimulus set, large pitch contrasts exist between the statements and questions (Patel et al., 1998), and research has shown that even individuals with congenital amusia, a neurodevelopmental disorder of pitch processing, can perform as well as TD controls on both identification and discrimination of these statements/questions (Ayotte et al., 2002; Patel et al., 2008). Addressing the issue with ceiling performance in the literature, Liu et al. (2010) designed and created a new set of ecologically valid stimuli with relatively subtle pitch contrasts between statements and questions, and revealed prosodic deficits in congenital amusia. Thus, using stimuli from Liu et al. (2010), the current study aimed to examine whether English-speaking individuals with ASD would also show impaired statement-question identification and discrimination when task difficulty is increased.

In addition to identification/discrimination accuracy rates, it has been suggested that participants’ response patterns should also be scrutinised in order to detect possible response biases in ASD (Järvinen-Pasley et al., 2008a). Specifically, Peppé et al. (2007) observed that while children with ASD performed as well as controls in terms of judgement accuracy in statement-question identification, they were biased towards judging questions as statements. In this study, 12.9% of the ASD participants and 2.7% of the control participants judged all the questions as statements, showing a declarative bias, although this percentage difference did not reach statistical significance (Peppé et al., 2007). For discrimination, impaired performance in ASD was mainly driven by false alarms, i.e. judging the same items as different (Peppé et al., 2007). To investigate the declarative bias in ASD further, Järvinen-Pasley et al. (2008a) examined another sample of participants and included the identification task in Patel et al. (1998) in addition to the turn-end task in PEPS-C. While no significant group difference in response patterns was observed for the turn-end task in PEPS-C, a significant declarative bias was observed among 50% of participants with ASD (in comparison to 10% of controls) for the identification task from Patel et al. (1998) (Järvinen-Pasley et al., 2008a). However, no response bias emerged among Mandarin speakers with ASD for either identification or discrimination in Jiang et al. (2015), although significantly lower accuracy rates were observed in ASD compared to TD. Thus, among the studies that examined response biases in ASD, mixed findings have been presented, with some studies indicating a response bias based on either statistics or simply percentage comparison (Järvinen-Pasley et al., 2008a; McCann et al., 2007; Peppé et al., 2007), whereas others reporting no response bias (Jiang et al., 2015), depending on the tasks and samples.

In summary, despite much research (Chevallier et al., 2009; Filipe et al., 2014; Järvinen-Pasley et al., 2008a; Jiang et al., 2015; McCann & Peppé, 2003; McCann et al., 2007; Paul et al., 2005; Peppé et al., 2007), it remains unclear whether individuals with ASD are associated with deficits in identification and/or discrimination of statements and questions, and whether there are response biases driving the observed accuracy rates. These questions need to be addressed, as the answers have implications for the prosody phenotypes of ASD. As mentioned earlier, due to the limitations of the design in PEPS-C (Peppé & McCann, 2003), the short-item discrimination task contains not only statement-question pairs but also liking-disliking pairs, and in laryngographic sounds rather than in natural speech. Thus, one cannot make inferences about the ability to discriminate statements from questions in everyday language from this task. However, if it is indeed the case that ASD is associated with intact identification but impaired discrimination as reported in Peppé et al. (2007), this dissociation between identification and discrimination may be interpreted as a special feature related to ASD phenotypes (Peppé et al., 2007). An association between identification and discrimination has been observed in other studies: both are intact (Järvinen-Pasley et al., 2008a); or both impaired (Jiang et al., 2015). To further clarify this issue and to help understand the phenotypes of ASD, the current study employed both identification and discrimination tasks from Liu et al. (2010) to investigate response patterns and the relationship between statement-question identification and discrimination in ASD.

### Production of Statement-Question Intonation in ASD

In contrast to the mixed findings reported in perception studies, evidence from production studies has consistently suggested atypical intonation production in ASD (Filipe et al., 2014; Fusaroli et al., 2017; McCann & Peppé, 2003; McCann et al., 2007). Specifically, statement responses of individuals with ASD were more likely to be judged as questions or ambiguous than those of controls (McCann et al., 2007; Peppé et al., 2007). In addition, utterances by individuals with ASD were much less likely to be judged as normal or natural than those of controls (Filipe et al., 2014). These ratings were either given by the experimenter (“tester”) (McCann et al., 2007; Peppé et al., 2007) or by independent adult participants (Filipe et al., 2014). Although informative, subjective ratings do not reveal what aspects of intonation production were atypical in ASD (e.g. pitch, duration, and intensity). In studies using objective acoustic measures, individuals with ASD showed significantly greater pitch range, mean pitch, and maximum pitch than controls for both statements and questions (Filipe et al., 2014), and increased and inappropriate use of pitch accents as well as difficulty in producing high frequency boundary tones (Fosnot & Jun, 1999). These findings were supported by Fusaroli et al. (2017), who systematically reviewed the literature quantifying acoustic patterns in ASD and identified significant differences in pitch production (e.g. pitch range and mean pitch) between individuals with ASD and controls, while finding no significant differences in other acoustic features (e.g. intensity, duration). In sum, the atypical production of intonation in ASD seems to be related to a salient acoustic parameter—pitch (DePape et al., 2012; Fusaroli et al., 2017).

However, previous studies on intonation production in ASD (Filipe et al., 2014; Fosnot & Jun, 1999; McCann et al., 2007; Peppé et al., 2007) have not conducted acoustic analysis to verify the acoustic realisation of pitch direction in statements and questions in ASD. Acoustic measures are important because question and statement intonation are heavily dependent upon pitch direction, with rising tones representing questions and falling tones representing statements (Cruttenden, 1997; Lieberman, 1960). Misuse of pitch itself can cause not only atypical intonation production but also misperception of statements and questions. Indeed, studies on congenital amusia have demonstrated the importance of acoustic analysis in quantifying pitch realisation in production, when examining the relationship between production and perception (Hutchins & Peretz, 2012; Liu et al., 2010; Loui et al., 2008). However, it remains to be determined whether intonation production and perception abilities are related or dissociated among individuals with ASD. The current study addressed this issue by including an intonation imitation task and using acoustic measures to assess pitch direction of the final words in the produced statements and questions (Liu et al., 2010).

### Perception of Pitch in Speech Versus Music in ASD

As in speech, pitch is also used extensively in music to convey meaning and emotion (Patel, 2008). It has been intensely debated whether pitch processing is domain-specific or domain-general between speech and music domains (Mantell & Pfordresher, 2013; Patel, 2008; Peretz & Coltheart, 2003). In particular, Peretz and Coltheart (2003) proposed that pitch information within a musical context is processed by a tonal encoding module which is absent in spoken pitch processing. Other researchers, however, argued for shared systems underlying the processing of information across both domains (Koelsch, 2011; Koelsch & Siebel, 2005; Patel, 2008; Sammler et al., 2009). Comparing intonation perception with melodic contour perception, Jiang et al. (2015) observed enhanced/intact melodic contour identification/discrimination but impaired statement-question identification and discrimination in Mandarin speakers with ASD. This finding suggested pitch processing deficits specific to the speech domain in ASD (Jiang et al., 2015). However, other studies indicated enhanced identification of pitch contours (e.g. rising, falling, falling-rising, rising-falling) across speech and musical stimuli (Järvinen-Pasley et al., 2008b), as well as superior discrimination of pitch patterns across speech-speech and speech-music stimulus pairs in ASD versus TD (Järvinen-Pasley & Heaton, 2007). Therefore, further research is warranted to clarify the domain specificity or generality of pitch processing in ASD. To our knowledge, no studies have yet compared pitch perception in ASD using speech and musical stimuli that are matched in global pitch contours derived from statement-question intonation. The present study aimed to fill this gap by investigating whether individuals with ASD would process intonation embedded in speech and musical stimuli differently, using the musical analogues of the statement-question discrimination task in Liu et al. (2010).

### The Development of Prosodic Abilities and its Relationship With Pitch Sensitivity

Studies of prosodic development in TD children suggest that there are significant improvements in the perception and production of statement-question intonation between ages 5 and 11 (Wells et al., 2004). As children grow older, pitch becomes the primary cue for the statement-question contrast compared to intensity and duration in production (Patel & Grigos, 2006). While 4-year-olds used lengthened duration of the final syllable rather than a rising pitch contour to signify questions, 7-year-olds used multiple acoustic cues (including pitch, intensity and duration) and 11-year-olds used pitch cues predominantly to differentiate statements from questions (Patel & Grigos, 2006). Given that language delay and impairment are prevalent among children and youth with ASD (Kwok et al., 2015), it may be the case that the development of prosodic skills is also delayed in ASD. Lyons et al., (2014) investigated the developmental changes of four prosodic functions, including the perception and production of statement-question intonation, stress, phrasing, and affect, in “language-normal” and “language-impaired” preadolescents (9–12 years old) and adolescents (13–17 years old) with and without ASD. The results suggest that TD preadolescents performed as well as TD adolescents on statement-question identification and production, and thus no developmental improvement was observed among TD participants due to ceiling performance. The same pattern of results was also seen in “language-normal” ASD preadolescents and adolescents, who performed similarly to the TD groups on both identification and production of statements and questions. For the “language-impaired” ASD groups, however, significant age-related improvement was observed for identification, but not for production, of statements and questions. That is, while impaired statement-question identification was only observed among “language-impaired” ASD preadolescents, but not among adolescents, impaired statement-question production persisted among “language-impaired” ASD preadolescents and adolescents. Thus, there are developmental delays in the perception and production of statements and questions among “language-impaired” individuals with ASD (Lyons et al., 2014).

In addition to the close relationship with language abilities (Lyons et al., 2014), prosodic skills also correlate significantly with pitch processing abilities (Liu et al., 2010, 2012; Vuvan et al., 2015). In typical development, there are age-related improvements in the ability to discriminate the direction of pitch changes between ages 6–11 (Fancourt et al., 2013). However, it has been reported that individuals with ASD show enhanced pitch discrimination early in development, and this ability maintains across children, adolescents and adults and does not correlate with receptive vocabulary (Mayer et al., 2016). By contrast, controls show significant gains in pitch discrimination performance across development, which also correlates significantly with receptive vocabulary scores (Mayer et al., 2016). This raises the questions as to whether and how pitch processing abilities influence intonation perception and production in individuals with ASD, and whether age plays a role in these abilities across the lifespan. The current study addressed these questions by examining the development of statement-question perception and production across children, adolescents and adults with and without ASD, as well as its relationship with pitch direction discrimination thresholds.

### Present Study

In the current study, we matched ASD and TD children, adolescents and adults for age, sex, nonverbal IQ, receptive vocabulary, as well as verbal and nonverbal short-term memory. Focusing on the prosodic feature of statement-question intonation and the acoustic parameter of pitch, we examined intonation processing in ASD and TD from the perspectives of task condition (discrimination, identification, imitation), response bias, stimulus type (speech, music), developmental changes, and its association with pitch thresholds. We asked whether individuals with ASD differed from controls in their ability to discriminate, identify, and imitate statement-question intonation, whether individuals with ASD showed response bias in discrimination and identification tasks, and whether performance on intonation perception and production related to pitch direction discrimination thresholds. We also examined whether individuals with ASD would perform better on musical pitch processing than on linguistic pitch processing, comparing discrimination of natural speech and their musical analogues. Finally, we examined the effect of age on pitch and intonation perception and production for both ASD and control groups. Based on previous findings, we predicted that: (a) participants with ASD would show impaired performance compared to controls in intonation discrimination and identification tasks, and they would show response biases towards judging the same pairs as different and identifying questions as statements; (b) participants with ASD would show poorer performance on the imitation task compared with controls; (c) participants with ASD would perform better on the musical condition than the speech condition in the discrimination task; (d) across both groups, performance on intonation processing would be associated with pitch direction discrimination thresholds; and (e) participants with ASD would show different developmental trajectories for pitch and intonation processing compared with controls.

## Methods

### Participants

A priori power analysis was conducted using G*Power (Faul et al., 2009). To detect the interaction of Group (ASD vs. control) by Condition (speech vs. music or identification vs. imitation) by Age (child, adolescent vs. adult) in the present design, 64 participants (with 32 in each group) were required to reach a power of 0.80, with a large effect size (f = 0.40) and an alpha of 0.05. Given the mixed findings in the ASD literature and to further increase the power of our study, we recruited a total of 84 participants, 42 with ASD (12 female, 30 male) and 42 controls (12 female, 30 male), resulting in a power of 0.91.

All participants were native speakers of British English, recruited through email lists, word of mouth, online and social media advertisements, local schools, charities and organisations, as well as departmental participant databases. Participants in the ASD group all received a formal diagnosis of ASD by professional clinicians (verified by official clinical reports), and their high autistic traits were also confirmed using the cut-off scores of 32 (adults), 30 (adolescents) and 76 (children) on the Autism-Spectrum Quotient (AQ) (Auyeung et al., 2008; Baron-Cohen et al., 2001, 2006). All control participants scored below these cut-offs. In addition to the AQ, Empathy Quotient (EQ) and Systemizing Quotient (SQ) were also collected through questionnaires. All participants had normal hearing in both ears, with pure-tone air conduction thresholds of 25 dB HL or better at frequencies of 0.5, 1, 2, and 4 kHz, as assessed using an Amplivox manual audiometer (Model 116). The study was approved by the University of Reading Research Ethics Committee. Written informed consent/assent was obtained from the participants and/or their parents prior to the experiment.

Given the significant effects of IQ, receptive vocabulary, short-term memory, and musical training on pitch and prosodic processing (Acton & Schroeder, 2001; Bidelman et al., 2013; Chowdhury et al., 2017; Heaton et al., 2008; Mayer et al., 2016; McCann et al., 2007; Peppé et al., 2007; Tillmann et al., 2016), we gathered related background measures from all participants (Table 1). Specifically, participants completed a nonverbal IQ test using the Raven’s Standard Progressive Matrices Test (Raven et al., 1998) and a receptive vocabulary test using the Receptive One Word Picture Vocabulary Test IV (ROWPVT-IV) (Martin & Brownell, 2011). The Corsi block-tapping task was used to assess participants’ nonverbal short-term memory span (Kessels et al., 2000), and the digit span task was used to assess verbal short-term memory (Wechsler, 2003). Participants’ musical training background was collected using a questionnaire, and their years of formal musical training were summed across all instruments including voice (Pfordresher & Halpern, 2013).

**Table 1:** Characteristics of the ASD (N = 42) and control groups (N = 42)

Age group	Diagnostic group	Age	Musical training	NVIQ	ROWPVT-IV	Corsi	Digit span	AQ	EQ	SQ
Children (N = 28)	ASD	9.42(1.19)	1.43(2.26)	73.93(26.11)	125.57(11.47)	4.79(0.80)	5.33(1.26)	95.86(23.18)	19.14(5.87)	29.71(10.88)
	control	9.35(1.44)	1.29(0.99)	83.57(20.80)	123.36(11.13)	5.29(1.27)	5.64(0.93)	47.77(19.70)	39.00(9.26)	26.77(4.40)
	Comparison statistics: Bayesian									
	W	94.5	115	113	67	112.5	104	13	171.5	73
	BF_01_	2.84	2.48	2.39	2.02	2.10	2.44	**0.05**	**0.04**	2.18
	Median	0.003	− 0.14	− 0.17	0.28	− 0.24	− 0.15	1.20	− 1.20	0.21
	95%CI	[− 0.64,0.66]	[− 0.83,0.52]	[− 0.90,0.51]	[− 0.36,1.00]	[− 0.99,0.42]	[− 0.90,0.52]	[0.34,2.10]	[− 2.05, − 0.33]	[− 0.45,0.93]
Adolescents (N = 20)	ASD	13.95(1.38)	3.40(3.06)	55(31.00)	116.2(18.71)	5.7(1.57)	5.7(1.06)	37.10(5.69)	13.00(6.67)	47.60(11.72)
	control	13.77(1.09)	3.10(3.00)	77(20.17)	134.3(12.46)	6.2(1.40)	6.2(0.79)	15.40(7.31)	45.63(12.46)	34.88(13.94)
	Comparison statistics: Bayesian									
	W	47.5	45	74	78	60	68	0	40	9
	BF_01_	2.43	2.21	**0.78**	**0.64**	2.10	1.60	**0.06**	**0.27**	1.07
	Median	0.03	0.15	− 0.61	− 0.66	− 0.19	-0.34	1.36	− 1.13	0.53
	95%CI	[− 0.73,0.74]	[− 0.57,0.95]	[− 1.55,0.17]	[− 1.61,0.14]	[-0.98,0.53]	[− 1.23,0.42]	[0.32,2.49]	[− 2.57,0.00]	[− 0.39,1.71]
Adults (N = 36)	ASD	35.47(13.07)	4.50(6.50)	50.00(28.54)	111.47(13.74)	5.72(1.49)	7.00(1.72)	35.83(9.21)	22.89(9.85)	79.17(28.55)
	control	35.34(12.88)	5.14(7.03)	41.94(29.06)	108.78(13.61)	6.06(1.00)	7.06(1.11)	15.06(6.53)	48.22(13.74)	49.89(16.31)
	Comparison statistics: Bayesian									
	W	162.5	158.5	135.5	143.5	190.5	171	15	307.5	65.5
	BF_01_	3.01	2.93	2.53	2.81	2.30	2.84	**0.01**	**0.01**	**0.11**
	Median	0.05	0.04	0.16	0.13	− 0.23	− 0.08	1.35	− 1.31	0.84
	95%CI	[− 0.55,0.62]	[− 0.54,0.65]	[− 0.44,0.81]	[− 0.44,0.76]	[− 0.86,0.38]	[− 0.71,0.53]	[0.58,2.15]	[− 2.06, − 0.55]	[0.17,1.55]

Age and Musical training are in years; NVIQ and ROWPVT-IV are percentile points of nonverbal IQ and standard scores of receptive verbal ability respectively; Corsi and Digit span are the raw scores of nonverbal and verbal short-term memory respectively; AQ, EQ and SQ are the scores of Autism Spectrum, Empathy and Systemizing Quotient respectively. Bayes factors from a default prior 2-tailed Bayesian Mann–Whitney-Wilcoxon Test are expressed in terms of the Bayes factor in favour of the null hypothesis of no difference (BF_01_). The delta effect size in these Bayesian comparisons is given by the median of *a posterior* distribution and 95% credible intervals

Following the age cut-offs for the Autism-Spectrum Quotient (Auyeung et al., 2008; Baron-Cohen et al., 2001, 2006), participants were divided into three age cohorts: children (7–11 years), adolescents (12–15 years), and adults (> = 16 years). The age range of the child cohort was between 7.39 and 11.92 years, that of the adolescent cohort was between 12.08 and 15.75, and that of the adult cohort was between 18 to 55.72 years. Table 1 shows the characteristics of the participants. The groups in each of the three age cohorts were largely matched on the background measures, with the exception that the ASD adolescents showed lower receptive vocabulary and nonverbal IQ scores than the control adolescents. To control for the possible contribution of receptive verbal ability and nonverbal IQ to the current results, these scores were entered as covariates in the analysis of Bayesian ANCOVA in the Results section.

### Tasks

The present study consisted of one pitch direction discrimination thresholds task and four intonation perception/production tasks from Liu et al. (2010). All tasks were conducted in a sound-proof booth at a university laboratory.

### Pitch Direction Discrimination Thresholds Task

Pitch thresholds were measured using an adaptive-tracking procedure with a “two down, one up” staircase method in Matlab (MATLAB, 2010). In each trial, participants were presented with three gliding tones of 600 ms each, centring on 500 Hz, with two moving in the same direction and the other moving in the opposite direction. Participants were required to identify the “odd-one-out” among the three tones. Starting with six semitones, the initial step size was one semitone which reduced to 0.1 semitones after four reversals and 0.02 semitones after eight reversals, with a total of 14 reversals. The threshold was calculated as the mean excursion size of the target glide of the last six reversals.

A practice session of four trials was provided to ensure that all participants understood the task. Participants were required to achieve 100% correct on the practice trials (with feedback) before proceeding to the testing session. Given that inattention may impact performance on adaptive-tracking pitch thresholds tasks, especially in children (Fancourt et al., 2013; Horváth et al., 2009; McDermott & Oxenham, 2008), participants were required to make their responses orally for the experimenters to input into the computer, in order to maintain their attention.

### Intonation Tasks

The intonation tasks consisted of four subtests assessing discrimination of statements and questions in natural speech and in their musical analogues (composed of gliding tones), and identification and imitation of these statements and questions. The tasks were presented in counterbalanced order across participants using Praat. Taken from Liu et al. (2010), the speech stimuli were 18 statement-question pairs, cross-spliced so that each pair began with the same stem and differed only in the final word (See Supplementary Figure S1 for examples, and Supplementary Table S1 for full details of the speech stimuli).

Musical analogues of the sentences were created in Praat (Boersma & Weenink, 2001), matching the original sentences in pitch and temporal patterns, following the procedure in Patel et al. (1998). These musical tones were made of the fundamental frequency and its seven odd harmonics of the individual syllables in the original sentences, with peak amplitudes normalized to match those of the sentences (see Liu et al. (2010) for full details).

The speech and musical discrimination tasks were conducted in two separate blocks (order counterbalanced), where participants were presented with 36 pairs of stimuli (either speech or musical analogues) in either the same or different condition. Participants were asked whether the pairs in each trial were the same or different, with their answers recorded by an experimenter by clicking a button on the computer. The interstimulus interval was 750 ms and the intertrial interval was 2 s. Two additional pairs were included as practice trials to familiarise participants with the procedure.

The identification and imitation tasks were conducted in the same block, where participants were presented with the 36 speech sentences one at a time. They were instructed to first imitate the sentence just played as exactly as possible (while their voices were recorded), and then to indicate whether the original sentence (not their imitation) was a statement or a question. The experimenter recorded the identification responses in Praat. Prior to the experiment, participants were familiarised with the procedure using two additional sentences (one statement and one question) in a practice session.

### Data Analysis

In the pitch direction discrimination task, thresholds were transformed using log transformation for parametric statistical analysis (Howell, 2009). Nine ASD participants and two controls did not complete this task. Additionally, to screen for inattentive performers, following Moore et al. (2008), the visual tracks were closely inspected on an individual basis. Two children were identified as “non-compliant” (a term used in previous literature; Fancourt et al. (2013)) performers due to fluctuations of attention, and their data were excluded from further analysis (see Supplementary Figure S2 for their visual tracks and Supplementary Table S2 for the remaining participants’ demographic characteristics in the pitch direction discrimination task).

Signal detection analysis was carried out for the intonation data. Specifically, in the discrimination tasks, correct responses to “different” trials were coded as hits; and in the identification and imitation tasks, correct responses to “question” trials were coded as hits, and *d'* was corrected using the log-linear rule (Hautus, 1995). For the recordings from the imitation task, we employed acoustic analysis as a quantitative measure of performance accuracy using an earlier version of ProsodyPro (Xu, 2013) in Praat. Signed glide sizes (in Hz) were extracted from the final words of the sentences, with negative values indicating downward glides and recorded as statements, and positive values indicating upward glides and recorded as questions (Liu et al., 2010).

Bayesian analyses were run using JASP software (JASP Team, 2020). Bayes Factors indicate the strength of the evidence obtained and are particularly helpful in determining when the evidence supports the null hypothesis over an alternative. Unlike frequentist statistics, Bayes Factors test the relative probability of the two hypotheses given the data, rather than the probability of the data given the null hypothesis and so can be used to support both alternative and null hypotheses (Dienes, 2014). The Bayes Factor (BF) in favour of the null is the reciprocal of the BF in favour of the alternative. Also unlike frequentist significance testing, BFs give continuous measures of the likelihood of one hypothesis over another, which means cut-off values (e.g. *p* = 0.05) are inappropriate. For the interpretation of BFs as evidence for hypotheses, Raftery (1995, p.139) suggested ranges of values equivalent to different “strengths” of evidence, where a BF value above 1 and less than 3 is “*weak*” evidence and a BF between 3 and 20 represents “*positive*” evidence for a hypothesis.

## Results

### Pitch Direction Discrimination Task

**Fig. 1:**
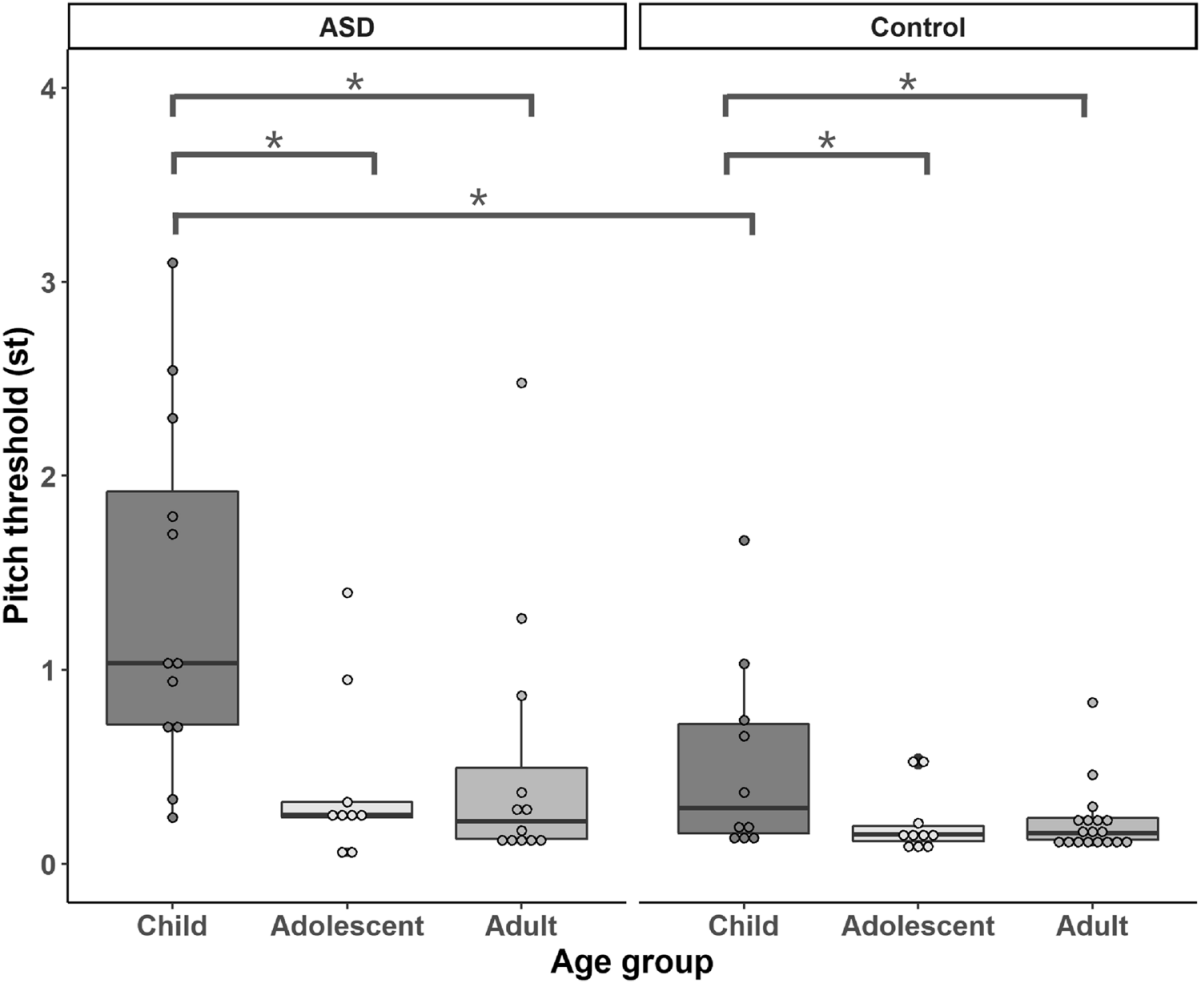
Pitch threshold in semitone (st) of each age cohort by group from the pitch direction discrimination task

Figure 1 shows boxplots of the pitch thresholds for the ASD and control groups. Bayesian ANCOVA was fit to the data with group (ASD vs. control) and age cohorts (child, adolescent and adult) as the between-subjects variables and receptive vocabulary and nonverbal IQ scores as the covariates. The model revealed positive main effects of age (BF_10_ = 400,381.43) and group (BF_10_ = 23.85) on pitch thresholds. The interaction between age and group also received positive support from Bayesian factor (BF_10_ = 4.70). The Bayesian post-hoc analysis suggested that both groups showed similar developmental trajectories, with the adult and adolescent cohorts performing better than the child cohort, though this trend was more pronounced in the ASD group than in the control group (see Table 2). The main effect of group was mainly driven by the difference across the child cohorts, as ASD children showed worse pitch thresholds than control children (BF_10_ = 7.07). The difference between the adult cohorts was weak (BF_10_ = 1.79) and no difference was found between the adolescent cohorts (BF_10_ = 0.46, *weak* evidence in favour of H_0_).

**Table 2:** Differences of pitch thresholds between age cohorts within each group

		ASD	Control
Adolescent	Child	BF_10_ = 15.00	BF_10_ = 1.52
Adult	Child	BF_10_ = 14.97	BF_10_ = 2.54
Adult	Adolescent	BF_10_ = 0.40	BF_10_ = 0.38

There was a main effect of nonverbal IQ (BF_10_ = 251), whereas the main effect of receptive verbal ability was only weakly supported by Bayes factor (BF_10_ = 1.82). A Bayesian Kendall correlation analysis (1-tailed) showed that nonverbal IQ and receptive verbal ability were weakly associated with the performance of the control group (NVIQ: tau = − 0.25, BF_-0_ = 1.14; ROWPVT-IV: tau = − 0.18, BF_-0_ = 1.30) but not those of the ASD group (NVIQ: tau = − 0.14, BF_-0_ = 0.74; ROWPVT-IV: tau = − 0.04, BF_-0_ = 0.30).

### Intonation Discrimination Tasks

**Fig. 2:**
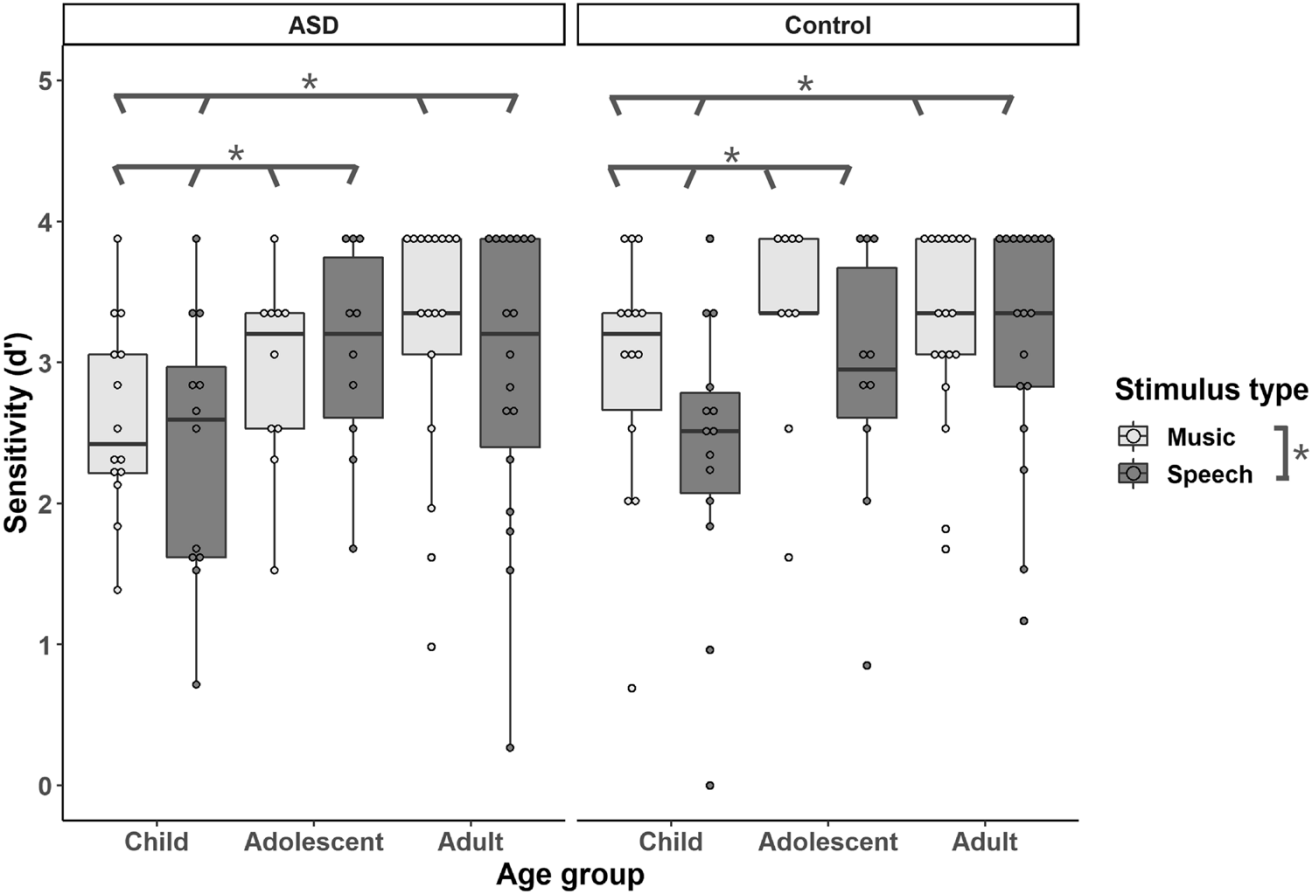
*d*' of each age cohort by stimulus type and by group from the discrimination task

Figure 2 shows boxplots of the sensitivity d’ on the discrimination tasks for the ASD and control groups. Bayesian repeated measures ANCOVA was fit to the data with group (ASD vs. control) and age cohorts (child, adolescent and adult) as the between-subjects variables, stimulus type (speech vs. music) as the within subject-variable, and receptive vocabulary and nonverbal IQ scores as the covariates. The model revealed a positive main effect of stimulus type (BF_10_ = 7.09), with all groups performing better on the music condition than on the speech condition, and a main effect of age (BF_10_ = 3.57). The Bayesian post-hoc analysis showed that the adult cohort performed better than the child cohort (BF_10_ = 47.19), and the adolescent cohort also performed better than the child cohort (BF_10_ = 2.95), whereas the adult and adolescent cohorts performed comparably (BF_10_ = 0.28, *positive* evidence in favour of H_0_). No evidence for other main effects or interactions was observed, though the evidence for the null hypotheses was also weak: group (BF_10_ = 0.35), stimulus type by age cohort (BF_10_ = 0.59), stimulus type by group (BF_10_ = 0.58), age cohort by group (BF_10_ = 0.35) and stimulus type by age cohort by group (BF_10_ = 0.34). The evidence for main effects of receptive verbal ability (BF_10_ = 0.995), and nonverbal IQ (BF_10_ = 0.91), was equivocal.

To assess the relationship between pitch thresholds and the performance on intonation discrimination, 1-tailed Bayesian Kendall’s correlation analysis was carried out separately for the groups and tasks, results are reported in Table 3. Pitch thresholds were negatively correlated with performance on both tasks for both groups: the lower (better) the pitch thresholds, the better performance on the speech and musical tasks.

**Table 3:** Kendall’s correlations between performance on pitch thresholds and intonation discrimination tasks by group

	ASD group	Natural speech	Musical analogues	Control group	Natural speech	Musical analogues
Pitch threshold	tau	− 0.33	− 0.34	tau	− 0.38	− 0.45
	BF_-0_	11.88	15.60	BF_-0_	91.05	765.98

### Intonation Identification and Imitation Tasks

**Fig. 3:**
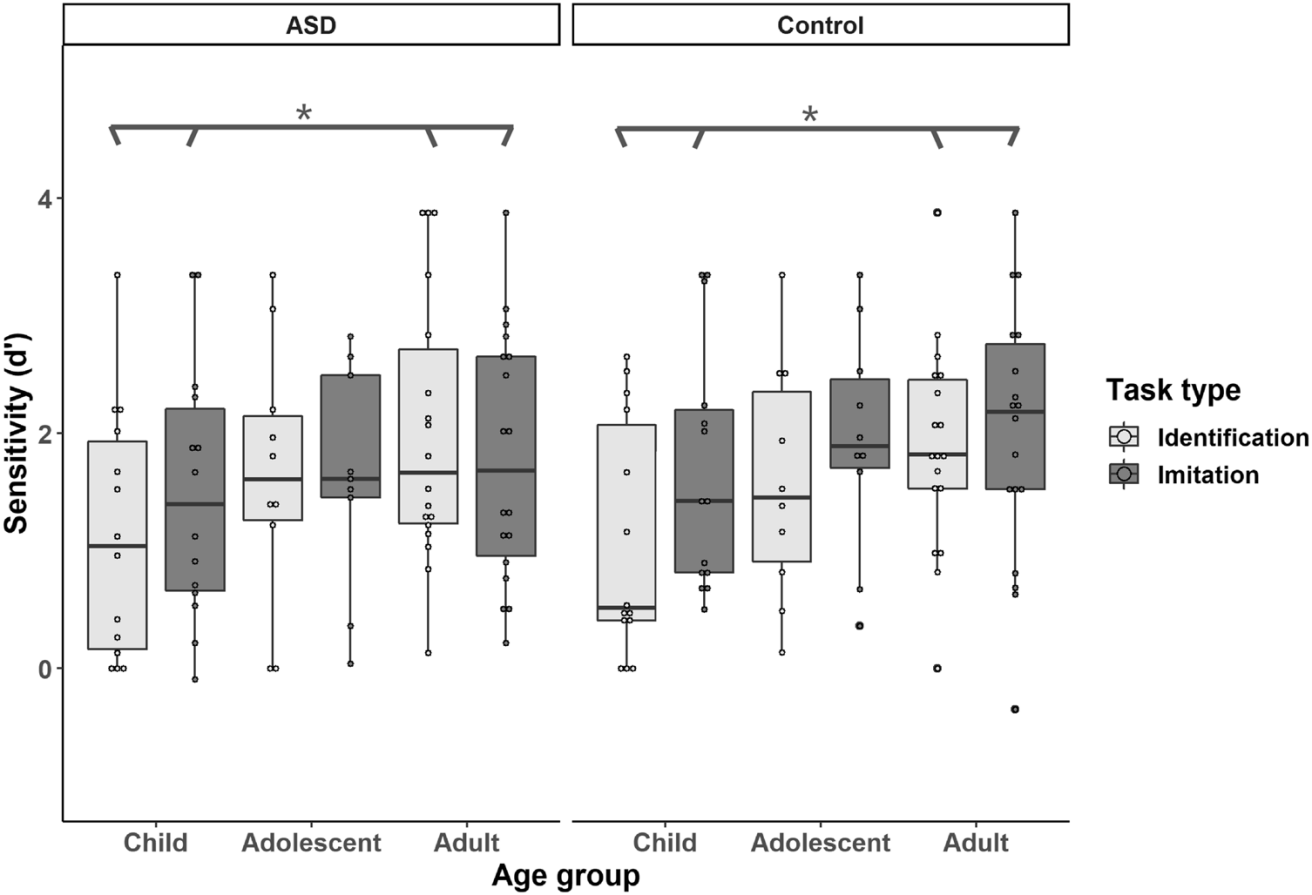
*d*' of each age cohort by group from the identification and imitation task

Figure 3 shows boxplots of the sensitivity d’ on the identification and imitation tasks for the ASD and control groups. A Bayesian repeated measures ANCOVA was conducted with group (ASD vs. control) and age (child, adolescent and adult) as the between-subjects variables, task type (identification vs. imitation) as the within-subject variable, and receptive vocabulary and nonverbal IQ scores as the covariates. The model revealed a main effect of age (BF_10_ = 4.06). The post-hoc analysis showed that the adult cohort performed better than the child cohort (BF_10_ = 18.01) but similarly to the adolescent cohort (BF_10_ = 0.57), and the adolescent and the child cohorts performed comparably (BF_10_ = 0.47). For all other main effects and interactions, there was positive evidence in favour of the null hypotheses: task type (BF_10_ = 0.23); group (BF_10_ = 0.14); task type by age cohort (BF_10_ = 0.33); task type by group (BF_10_ = 0.13); age cohort by group (BF_10_ = 0.11) and task type by age cohort by group (BF_10_ = 0.01).

In addition, there was a weak main effect of receptive verbal ability, BF_10_ = 2.93, whereas no main effect of nonverbal IQ was observed, BF_10_ = 0.73. However, a Bayesian Kendall correlation analysis (1-tailed) showed that receptive verbal ability was not associated with performance on the imitation task (ASD: tau = 0.08, BF_+0_ = 0.40; control: tau = 0.10, BF_+0_ = 0.50) or the identification task (ASD: tau = 0.02, BF_+0_ = 0.23; control: tau = 0.07, BF_+0_ = 0.38) in either group, with Bayes factors supporting the null hypotheses positively.

Performance on identification and imitation was positively correlated for both groups (ASD: tau = 0.40, BF_+0_ = 329.97; control: tau = 0.28, BF_+0_ = 11.50). Using 1-tailed Bayesian Kendall’s correlation analysis, we analysed the relationship between pitch thresholds and identification as well as imitation tasks (see Table 4). The results indicated that pitch thresholds were negatively correlated with the performance on both tasks in the control group, but were only weakly associated with the identification performance in the ASD group.

**Table 4:** Kendall’s correlations between performance on pitch thresholds and intonation identification/imitation tasks by group

	ASD group	Intonation identification	Intonation imitation	Control group	Intonation identification	Intonation imitation
Pitch threshold	tau	− 0.22	− 0.15	tau	− 0.43	− 0.26
	BF_-0_	2.06	0.80	BF_-0_	509.47	5.09

### Response Bias

To measure whether individuals with ASD showed response biases between the same versus different pairs (discrimination task) or questions versus statements (identification task), i.e. judging the same pairs as different or questions as statements, we calculated the percentage of correct responses to same/different pairs in the discrimination task and questions/statements in the identification task for the two groups.

A 2 × 2 mixed ANOVA with Bayesian analysis was conducted. Group (ASD vs. control) was the between-subjects factor, and response type (same vs. different in the discrimination task and question vs. statement in the identification task) was the within-subjects factor. In the discrimination task, there was a main effect of response type (BF_10_ = 137.42) with participants showing poorer performance on different pairs (M = 0.90, SD = 0.12) than on same pairs (M = 0.94, SD = 0.07). No main effect of group, or group by response type interaction was found, with Bayes factors tending to support the null hypotheses in both cases (BF_10_ = 0.32 and BF_10_ = 0.52, respectively). In the identification task, there was a main effect of response type, BF_10_ = ∞, with participants showing poorer performance on questions (M = 0.62, SD = 0.22) than statements (M = 0.90, SD = 0.15). No main effect of group or group by stimulus type interaction was observed, with BF_10_ = 0.20 and BF_10_ = 0.24, respectively.

To inspect individual response patterns, following Steffens et al. (2020), we calculated the probability that each individual accuracy rate was due to random guessing based on the binomial distribution. Accuracy rates with probabilities > 0.05 were interpreted as being likely due to random chance alone, whereas accuracy rates with probabilities ≤ 0.05 were interpreted as being unlikely due to chance alone (Steffens et al., 2020). We found that all participants performed above chance level in the discrimination task, while 24 participants showed chance level performance in the identification task (12 ASD vs. 12 control). A 2 × 2 mixed ANOVA with Bayesian analysis with Group as the between-subjects factor and response type as the within-subjects factor on the responses of the 24 participants revealed no main effect of group, BF_10_ = 0.28, or group by stimulus type interaction, BF_10_ = 0.37. There was a main effect of stimulus type, as participants were less able to identify questions (M = 0.27, SD = 0.24) than statements (M = 0.82, SD = 0.20).

## Discussion

Using pitch thresholds and intonation perception and production tasks, the present study examined the abilities of individuals with and without ASD to use pitch to differentiate, identify, and imitate intonation (statements vs. questions) and whether these abilities would be affected by response bias, age (child, adolescent vs. adult), stimulus type (speech vs. music), and pitch direction discrimination thresholds. The main results showed that the performance of intonation discrimination (in both speech and music conditions), identification, and imitation was comparable between the ASD and TD groups within each age cohort, and that performances across tasks were largely independent of participants’ receptive verbal ability and nonverbal IQ, especially for participants with ASD. In addition, no response bias was observed in the discrimination and identification of statements and questions among participants with ASD. Participants’ abilities to discriminate, identify and imitation intonation were associated with their pitch direction discrimination thresholds for both groups. There were also age-related improvements across all tasks for both groups. These findings suggest that some individuals with ASD may have genuinely intact abilities to differentiate, identify, and imitate statement-question intonation, and they may also show similar developmental trajectories as typically developing individuals, with performance on both intonation and pitch thresholds increasing with age.

### Perception of Statement-Question Intonation and Response Bias in ASD

Regarding discrimination and identification of statements and questions, we found no group differences in response accuracy across all three age cohorts. Bayesian analyses supported our null results weakly for the discrimination task but positively for the identification task. Thus, no strong conclusions can be drawn about intonation discrimination abilities between the ASD and control groups. These findings are consistent with the majority of the literature that suggests intact statement-question identification in ASD (Chevallier et al., 2009; Filipe et al., 2014; Järvinen-Pasley et al., 2008a; Paul et al., 2005). However, they contradict the findings indicating impaired discrimination (McCann et al., 2007; Peppé et al., 2007), impaired identification among “language-impaired” preadolescents (Lyons et al., 2014), and impaired discrimination and identification among Mandarin speakers (Jiang et al., 2015). Notably, as previously mentioned, the impaired discrimination suggested by McCann et al. (2007) and Peppé et al. (2007) was evaluated using the short-item discrimination task within PEPS-C, which contains the laryngographic sounds of the statement-question pairs, as well as those of the liking-disliking pairs from the affect subtask. Thus, the discrimination performance in ASD reported by these two studies may be confounded by the unnaturalness of the stimuli as well as by participants’ ability to discriminate affective pairs.

In addition, the inconsistency between the present study and Jiang et al. (2015) may be explained by language differences. Jiang et al. (2015) used stimuli in Mandarin which is a tone language, while the present study used stimuli in English which is a non-tone language. It has been suggested that the perception of statement-question intonation in tone languages is complicated by the changes in tones, which convey lexical meaning (Jiang et al., 2015; Liu & Xu, 2005; Xu, 2005), resulting in the tasks in Jiang et al. (2015) being more difficult than the present study. Finally, impaired statement-question identification was only observed among “language-impaired” preadolescents in Lyons et al. (2014). Indeed, prosodic skills correlate significantly with language ability in ASD (McCann et al., 2007; Peppé et al., 2007), In our current study, there was a weak main effect of receptive verbal ability on intonation identification/imitation (although correlations were nonsignificant), but not on intonation discrimination, which may be because our ASD and TD participants were largely matched on a range of cognitive abilities (Table 1).

We also examined the response biases that were reported in some studies (Järvinen-Pasley et al., 2008a; Peppé et al., 2007). Inconsistent with those results, but consistent with the findings of Jiang et al. (2015), individuals with ASD in our study did not show a tendency to judge the same pairs as different or identify questions as statements and this null result receives substantial support from the Bayes factors. Similar to controls, our participants with ASD displayed poorer performance when discriminating different pairs than same pairs, and when identifying questions than statements. While the response bias in the discrimination task reported by Peppé et al. (2007) reached a significant level, the declarative bias in identification from the PEPS-C turn-end task lacked statistical support (Järvinen-Pasley et al., 2008a; Peppé et al., 2007). Using the speech stimuli from Patel et al. (1998), Järvinen-Pasley et al. (2008a) observed a significant declarative bias among 50% of participants with ASD (in comparison to 10% of controls) for the identification task. Following Jiang et al. (2015) and Steffens et al. (2020), the present study used ANOVA models to inspect participants’ response patterns, and found no response bias in either discrimination or identification tasks in ASD. It is worth noting that the difference in results between our study and previous studies is not due to our sample size being smaller. In fact, our ASD sample size is the largest among all these studies: 42 (our study), 31 (McCann et al., 2007; Peppé et al., 2007), 21 (Järvinen-Pasley et al., 2008a), and 17 (Jiang et al., 2015). Given the reproducibility problems in science (Begley & Ioannidis, 2015), further studies are needed to determine whether there are genuine response biases in intonation discrimination and identification in ASD.

### Production of Statement-Question Intonation in ASD

In the imitation task, participants with ASD showed comparable performance to controls. Although previous research has found a deficit in intonation production in ASD, either based on subjective perceptual judgements or objective acoustic measures (Filipe et al., 2014; Fosnot & Jun, 1999; McCann et al., 2007; Peppé et al., 2007, 2011), the current study did not observe this deficit and the balance of evidence provided by our Bayesian analyses is sufficient for us to be confident in our null results. This discrepancy mainly results from the different methods used in reporting/analysing production data among these studies. Unlike previous studies using subjective judgements of the sentences produced (Filipe et al., 2014; McCann et al., 2007; Peppé et al., 2007, 2011), we explored objective measures by calculating glide sizes to verify the acoustic realisation of pitch direction in statements and questions in ASD. Thus, imitations were scored as correct only if participants shared the same sign in glide size as the models (i.e. statements imitated as statements with final falls would have negative glide sizes, and questions imitated as questions with final rises would have positive glide sizes). Additionally, while the present study was inspired by previous acoustic studies suggesting the key role pitch plays in atypical intonation production in ASD (Filipe et al., 2014; Fosnot & Jun, 1999), we used a different acoustic analysis method than those studies, in order to capture the production of pitch direction specifically. That is, when calculating imitation accuracy, we did not consider mean pitch, pitch range, or other variables, which measured the characteristics of speech production rather than imitation accuracy per se (Filipe et al., 2014; Fosnot & Jun, 1999). Rather, we focused on using objective measures to gauge the relationship between identification and imitation. The results suggested that identification abilities positively correlated with imitation abilities in both groups and that the association was stronger in the ASD group than in the control group, which were substantially supported by Bayes factors. These findings are consistent with the finding of intact identification and production of turn-end sentences in previous studies (Järvinen-Pasley et al., 2008a; Peppé et al., 2007). Our correlation analysis further indicates that the more accurate the participants were on prosody perception and understanding, the better their performance on prosody production. Thus, an increase in receptive prosodic skills might result in amelioration of expressive prosodic disorder in ASD, and vice versa.

### Perception of Pitch in Speech Versus Music in ASD

The third aim of this study was to investigate intonation processing in speech versus music in ASD. The results showed that, like the controls, participants with ASD performed better on discriminating between musical glides than on speech utterances. These findings received positive support from our Bayesian analysis. While the better performance on music than on speech in the intonation task is consistent with our hypothesis for ASD, the same perceptual pattern was also noted in the control group. It has been suggested that semantic information might hamper controls’ performance due to their overly selective attention towards the content (Järvinen-Pasley & Heaton, 2007). Similarly, Bijou and Ghezzi (1999) have proposed a Behavior Interference Theory which states that typically developing children tend to focus on social stimuli (i.e. the human voice), whereas these stimuli do not easily obtain attention from children with ASD. In the present study, both groups showed semantic interference with perceptual processing and performed better on discrimination of musical analogues than natural speech. This perceptual pattern is consistent with the findings of Liu et al. (2010) for amusic participants, Cheng et al. (2017) for ASD and control participants, as well as Francis and Ciocca (2003) for typically developing English and Cantonese listeners. However, other studies reported mixed findings regarding the effect of stimulus type on intonation processing among amusic and control participants (Jiang et al., 2010; Liu et al., 2012; Patel et al., 2005, 2008). Further studies are required to tease apart the effects of stimulus type, perceptual acuity, and sensory preference on intonation processing among different participant groups.

Since no group difference was observed in discrimination of speech and musical stimuli, the current findings provide evidence for shared mechanisms of pitch processing between music and speech in both individuals with ASD and controls (Liu et al., 2010). Similar findings were also reported in Cheng et al. (2017). However, numerous previous studies have suggested enhanced musical processing in ASD compared to controls, including local music processing (Mottron et al., 2000), melodic contour identification (Jiang et al., 2015), as well as memory and labelling of musical tones and segmentation of chords (Heaton, 2003). Nevertheless, more recent studies reported comparable or even impaired musical processing in ASD versus TD (Jamey et al., 2019; Schelinski et al., 2017; Sota et al., 2018). Thus, with mixed findings in the literature (Järvinen-Pasley & Heaton, 2007; Järvinen-Pasley et al., 2008b; Jiang et al., 2015), the domain specificity or generality of pitch processing in ASD warrants further studies.

### The Development of Prosodic Abilities and its Relationship With Pitch Sensitivity

The fourth aim of our study was to examine the relationship between psychophysical pitch thresholds and intonation perception/imitation. We found that children with ASD had elevated pitch thresholds relative to their typically developing counterparts with substantial support from Bayesian factors. Adults with ASD performed worse relative to adult controls, and adolescents with ASD performed comparably to their controls in the pitch thresholds task. However, these findings received weak support from Bayesians. So it would be premature to draw strong conclusions specific to these two age cohorts. In addition, our results point to positive relationships between the pitch thresholds and intonation perception/imitation in both groups: the more sensitive to pitch, the better performance on intonation perception. This correlation was more pronounced in the control group suggested by Bayes factors. This finding is consistent with previous research showing an overall positive relationship between low-level and higher-level pitch processing (Germain et al., 2019). These findings likely reflect a bottom-up cascading in which the degree of low-level strength or impairment influences performance at later stages, such as language acquisition and communication (Bertone et al., 2010; Germain et al., 2019; Stevenson et al., 2014).

Finally, the major contribution of the current study relates to the effects of age on pitch thresholds and intonation processing. Both groups showed age-related improvements across all tasks with positive support from Bayes factors. In particular, adults consistently showed smaller pitch thresholds and better intonation perception and imitation than children, suggesting a developmental improvement in pitch perception and intonation processing. Interestingly, age-related changes across the lifespan from children to adolescents to adults were not identical across different tasks. Specifically, there were no significant differences in pitch thresholds and intonation discrimination between the adult and adolescent cohorts, who performed significantly better than the child cohort on those tasks. In terms of intonation identification and imitation, however, there was a gradual improvement from children to adolescents to adults, with no significant difference between adjoining age cohorts, but the adult cohort was significantly better than the child cohort. These findings suggest that pitch processing ability may improve with age, and that although important developments in the understanding and use of prosody continue during the school years (Cruttenden, 1985; Lyons et al., 2014; Wells et al., 2004), it is not yet adultlike for both ASD and TD participants.

Our finding of similar developmental changes in pitch discrimination ability across the ASD and control groups is incompatible with the markedly different developmental trajectories described by Mayer et al. (2016), where pitch discrimination ability increased with age in the control group but remained stable and enhanced across age cohorts in ASD. The discrepancy between the studies may be explained in several ways. First, there were differences in the paradigms used between the studies. The present study used an adaptive-tracking pitch threshold task to measure participants' pitch sensitivity starting with a default excursion size of six semitones, while Mayer et al. (2016) used stimulus pairs with either the same pitch or at a distance corresponding to 2, 3 or 6 semitones. Thus, the pitch variability of the stimuli used in their study was coarse, resulting in the task being easier than the one in the current study. Second, in Mayer et al. (2016), participants’ ages overlapped between the child cohort (between 6 years 11 months and 14 years 9 months) and the adolescent cohort (between 9 years 8 months and 16 years 5 months), with both cohorts including intellectually lower-functioning ASD individuals, while the adult groups were all intellectually high-functioning. In our study, in order to match groups for age, sex and cognitive capability, all participants with ASD were intellectually high-functioning individuals and our age cohorts were defined with adults >  = 16, adolescents between 12–15, and children between 7–11 years. Finally, the data in Mayer et al. (2016) were collected and combined from three separate studies, which may have also affected the results.

Furthermore, in contrast to preadolescents with ASD whose language abilities fall within the average range for their age, those with language impairment showed developmental delays in the perception and production of statements and questions (Lyons et al., 2014). Our finding of no group difference in intonation discrimination, identification and imitation across age cohorts is in line with the results from preadolescents and adolescents with ASD who had age-appropriate language in Lyons et al. (2014). While there are many different ways to categorize age cohorts (Ahmad et al., 2009; Nithyashri & Kulanthaivel, 2012), the present study followed the division methods in the Autism-Spectrum Quotient (Auyeung et al., 2008; Baron-Cohen et al., 2001, 2006). One limitation of using this three-way split is that the age differences are not fine-grained, which may not be sufficient to detect subtle developmental changes over time. Hence, while we observed developmental changes from children to adults in both ASD and TD, further studies are required to use more fine-grained classifications of age cohorts together with larger sample sizes, in order to map detailed developmental trajectories of pitch and intonation processing abilities in both groups.

### Implications of the Current Research Findings

The current study has a basic research focus with the aim of determining whether, and how, sensitivity to pitch and prosody is affected in a group of participants with ASD when compared to a matched control group. In doing so, however, the study is also directly investigating the responses of individuals with ASD to social stimuli, as speech is an inherently social act. As social stimuli can sometimes be somewhat aversive to children with ASD, possibly by virtue of their unpredictability, nonverbal children with ASD typically require hundreds of hours of generalized imitation training (e.g. object imitation, gross-motor imitation, oral-motor imitation and vocal imitation) and echoic/vocal mand training to improve their spoken language skills (Hampton & Kaiser, 2016). Even so, interventions are not always successful, not least because of other comorbidities in such children which may interfere with their ability to perceive, acquire, or reproduce speech (Tager-Flusberg & Kasari, 2013). For such children, our results have a number of consequences.

Firstly, our data confirm that, absent such comorbidities and assuming sufficient general cognitive capability, there is no a priori reason to suppose that speech processing and production mechanisms are impaired when actively processing speech and therefore the perceptual, cognitive and motor mechanisms are likely to be in place to support any speech-therapeutic programme which may be indicated. Secondly, there is no evidence for a dissociation between pitch processing in music and speech in our investigations. The data therefore support the use of musical stimuli (which may be less aversive to some individuals) as scaffolding for training in pitch-based discrimination and imitation for individuals who may be more attracted to music than to language. Note however, both that our sample were not representative of children with ASD and learning difficulties, and that appropriate translational studies to confirm the generalisability of training in pitch in music to perception and production of pitch in speech are beyond the scope of the current investigation. Thirdly, despite the generally equivalent performance across groups in our tasks—and the lack of a response bias which might otherwise complicate interpretations on some tasks—pitch discrimination thresholds are elevated in the ASD group relative to the control group. This identifies and highlights a particular perceptual problem in this group although, somewhat surprisingly, not one that impacted upon performance in other tasks despite the overall negative correlations between pitch threshold and intonation discrimination in both music and speech.

## Conclusion

In the present study, an experimental, acoustics-based approach was used to investigate perception and production of prosody in ASD, facilitating objective comparisons between the two modalities of intonation. In addition, we examined intonation processing in ASD and TD from the perspectives of response bias, task condition (discrimination, identification, imitation), stimulus type (speech, music), developmental changes, and its association with pitch thresholds. Our study revealed that intonation discrimination (in both speech and music conditions), identification and imitation abilities may be intact in some individuals with ASD across age cohorts (children, adolescents and adults), although children with ASD tend to have elevated pitch direction discrimination thresholds than their typically developing counterparts. The ASD and control groups also showed a similar developmental improvement in pitch thresholds, intonation discrimination and identification, as well as imitation. Furthermore, intonation identification and imitation are associated in both individuals with ASD and their peers, suggesting that improvements in intonation comprehension may also contribute to intonation production, and vice versa. We also found an association between low-level pitch threshold and high-level intonation processing across all participants, which reflects that the degree of strength or impairment in low-level pitch processing may influence performance on language acquisition and/or communication skills, whereas this bottom-up effect is more pronounced in controls than in individuals with ASD. In summary, our findings provide evidence for shared mechanisms in pitch processing between speech and music, as well as associations between low- and high-level pitch processing and between perception and production of pitch in prosody in individuals with and without ASD, who also show similar developmental trajectories for these abilities. Further studies with individuals with ASD from different cultures, particularly in other languages, would be helpful in obtaining a more comprehensive understanding of these shared mechanisms in ASD.

## Supplementary Information

Below is the link to the electronic supplementary material.

Supplementary file1 (docx 604 kb)
