# Supplementary material for: Perception and Production of Statement-Question Intonation in Autism Spectrum Disorder: A Developmental Investigation
Source: J Autism Dev Disord. 2021 Aug 5;52(8):3456–72. doi: 10.1007/s10803-021-05220-4 (PMC9296411; doi:10.1007/s10803-021-05220-4)
Supplement: Supplementary file 1 — Supplementary file1 (docx 604 kb) [file 10803_2021_5220_MOESM1_ESM.docx]

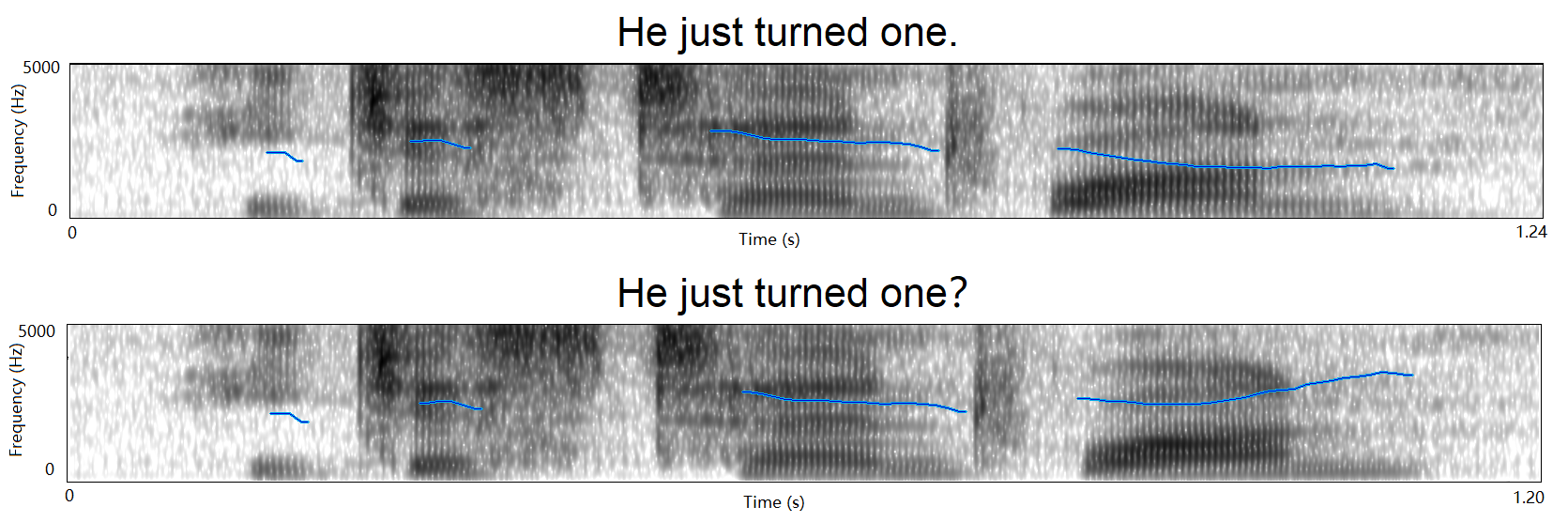
Supplementary Figure S1. Spectrograms of an example sentence pair “He just turned one./?” with different final pitch contours in the statement (falling) and the question (rising).


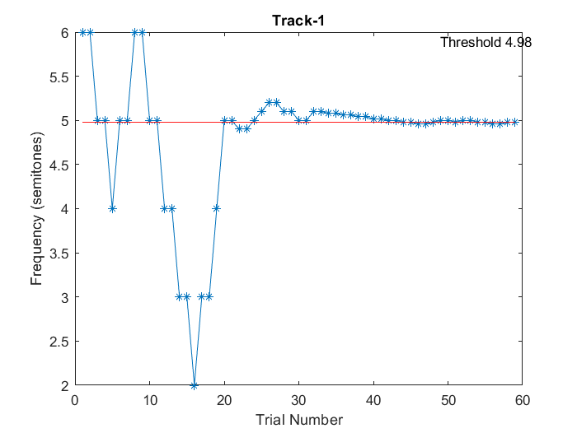

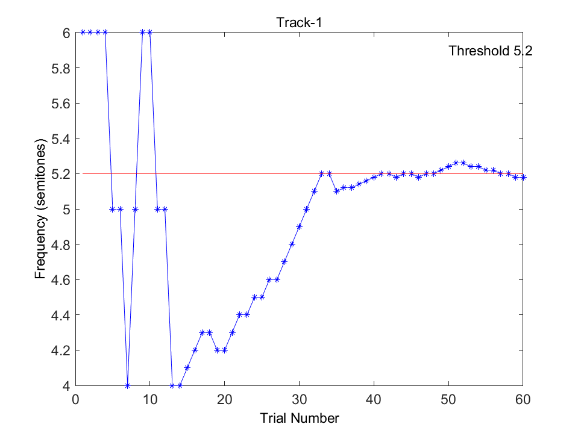


Supplementary Figure S2. Visual tracks of two non-compliant performers on the pitch direction discrimination task.

Supplementary Table S1. Sentences used in intonation tasks.

| Sentence | Sentence Rate (syl/s) | | Size of Final Pitch Glide (st) | | Rate of Final Pitch Glide (st/s) | | Duration of Final Pitch Glide (s) | |
| --- | --- | --- | --- | --- | --- | --- | --- | --- |
|  | S | Q | S | Q | S | Q | S | Q |
| It’s a lie./? | 4.7 | 4.4 | -2.5 | 7.1 | -11.3 | 40.4 | 0.22 | 0.18 |
| This is love./? | 3.9 | 3.5 | -3.6 | 2.6 | -17.4 | 20.7 | 0.21 | 0.12 |
| He hurt his knee./? | 4.3 | 4.2 | -3.0 | 3.7 | -16.7 | 21.6 | 0.18 | 0.17 |
| The answer is no./? | 5.6 | 5.6 | -2.7 | 4.7 | -24.3 | 28.3 | 0.11 | 0.17 |
| The deal is still on./? | 5.5 | 5.3 | -3.7 | 4.4 | -24.3 | 31.4 | 0.15 | 0.14 |
| He just turned one./? | 3.9 | 4.0 | -5.1 | 4.0 | -29.5 | 16.6 | 0.17 | 0.24 |
| She looks like Anne./? | 3.9 | 4.0 | -2.4 | 3.4 | -17.2 | 26.3 | 0.14 | 0.13 |
| She changed her name./? | 4.3 | 4.3 | -4.5 | 5.5 | -16.7 | 35.3 | 0.27 | 0.16 |
| It's a menu./? | 5.4 | 5.5 | -5.4 | 2.9 | -18.8 | 17.2 | 0.29 | 0.17 |
| She looks manly./? | 4.3 | 3.9 | -6.2 | 4.1 | -14.8 | 12.4 | 0.41 | 0.33 |
| He lives in Ealing./? | 5.7 | 5.6 | -6.4 | 8.1 | -27.1 | 32.6 | 0.24 | 0.25 |
| She grew up in Ely./? | 5.8 | 5.8 | -4.0 | 9.6 | -18.3 | 29.2 | 0.22 | 0.33 |
| They were in a limo./? | 6.7 | 6.4 | -3.5 | 6.9 | -17.5 | 40.7 | 0.20 | 0.17 |
| They named her Lilly./? | 5.3 | 5.4 | -4.2 | 4.9 | -25.5 | 26.5 | 0.17 | 0.18 |
| It’s from Emily./? | 6.1 | 6.1 | -4.1 | 5.0 | -26.3 | 12.7 | 0.16 | 0.39 |
| He speaks Romany./? | 4.6 | 4.4 | -3.3 | 2.9 | -7.5 | 10.3 | 0.44 | 0.28 |
| He was born in Illinois./? | 6.1 | 5.9 | -3.0 | 3.2 | -8.0 | 18.9 | 0.37 | 0.17 |
| He considers her his enemy./? | 6.7 | 6.5 | -3.7 | 4.8 | -10.4 | 19.3 | 0.35 | 0.25 |
| **Mean** | **5.1** | **5.0** | **-4.0** | **4.9** | **-18.4** | **24.5** | **0.24** | **0.21** |
| **SD** | **0.9** | **1.0** | **1.2** | **1.9** | **6.6** | **9.3** | **0.10** | **0.08** |

S = statement; Q = question; syl=syllable; st=semitone.

Supplementary Table S2. Characteristics of existing participants on the pitch direction discrimination task (N = 71).

| **Age group** | **Diagnostic group** | **Age** | **Music training** | | | | **NVIQ** | | | | **ROWPVT-IV** | | | **Corsi** | **Digit span** | **AQ** | **EQ** | **SQ** |
| --- | --- | --- | --- | --- | --- | --- | --- | --- | --- | --- | --- | --- | --- | --- | --- | --- | --- | --- |
| **Children**  ASD = 12  control = 10 | ASD | 9.28(1.23) | 1.54(2.42) | | | | 72.68(27.59) | | | | 123.00(10.11) | | | 4.67(0.99) | 5.42(1.00) | 96.58(24.55) | 19.42(6.30) | 30.08(11.21) |
|  | control | 9.71(1.48) | 1.50(0.97) | | | | 80.00(23.92) | | | | 124.80(12.79) | | | 5.50(1.43) | 5.80(0.79) | 44.20(19.60) | 39.90(9.70) | 26.40(4.86) |
|  | **Comparison statistics: Bayesian** | | |  | |  | | |  | | |  |  |  |  |  |  |  |
|  | W | 72.5 | 74.5 | | | | 66.5 | | | | 50 | | | 76 | 78 | 9 | 112 | 46 |
|  | BF_01_ | 1.96 | 2.11 | | | | 2.29 | | | | 2.46 | | | 1.58 | 1.75 | **0.12** | **0.16** | 2.06 |
|  | Median | -0.26 | -0.18 | | | | -0.11 | | | | 0.07 | | | -0.36 | -0.30 | 1.09 | -1.18 | 0.21 |
|  | 95%CI | [-1.06,0.42] | [-0.97,0.53] | | | | [-0.90,0.60] | | | | [-0.65,0.82] | | | [-1.23,0.37] | [-1.15,0.41] | [0.20,2.11] | [-2.18,-0.21] | [-0.49,1.02] |
| **Adolescents**  ASD = 9  control = 10 | ASD | 13.87(1.44) | 3.56(3.21) | | | | 51.11(30.19) | | | | 115.78(19.80) | | | 5.78(1.64) | 5.67(1.12) | 37.22(6.02) | 13.00(6.67) | 47.60(11.71) |
|  | control | 13.77(1.09) | 3.10(3.00) | | | | 77(20.17) | | | | 134.3(12.46) | | | 6.20(1.40) | 6.20(0.79) | 15.40(7.31) | 45.63(12.46) | 34.88(13.94) |
|  | **Comparison statistics: Bayesian** | | |  | |  | | |  | | |  |  |  |  |  |  |  |
|  | W | 44.5 | 39.5 | | | | 69.5 | | | | 69.5 | | | 52 | 62 | 0 | 40 | 9 |
|  | BF_01_ | 2.46 | 2.13 | | | | **0.58** | | | | **0.83** | | | 2.13 | 1.55 | **0.07** | **0.28** | 1.15 |
|  | Median | -0.002 | 0.16 | | | | -0.68 | | | | -0.59 | | | -0.16 | -0.36 | 1.29 | -1.13 | 0.51 |
|  | 95%CI | [-0.77,0.77] | [-0.60,0.94] | | | | [-1.67,0.14] | | | | [-1.55,0.22] | | | [-0.97,0.61] | [-1.28,0.43] | [0.28,2.43] | [-2.56,0.00] | [-0.36,1.69] |
| **Adults**  ASD = 12  control = 18 | ASD | 37.68(14.45) | 5.83(7.50) | | | | 50.00(32.12) | | | | 107.83(11.55) | | | 5.58(1.68) | 7.25(1.96) | 36.67(10.33) | 20.92(5.55) | 79.33(33.86) |
|  | control | 35.34(12.88) | 5.14(7.03) | | | | 41.94(29.06) | | | | 108.78(13.61) | | | 6.06(1.00) | 7.06(1.11) | 15.06(6.53) | 48.22(13.74) | 49.89(16.31) |
|  | **Comparison statistics: Bayesian** | | | |  | | |  | |  | | |  |  |  |  |  |  |
|  | W | 97.5 | 94 | | | | 94.5 | | | | 115.5 | | | 134 | 104 | 12 | 215 | 55 |
|  | BF_01_ | 2.45 | 2.41 | | | | 2.56 | | | | 2.76 | | | 1.86 | 2.66 | **0.03** | **0.02** | **0.44** |
|  | Median | 0.17 | 0.16 | | | | 0.10 | | | | -0.05 | | | -0.29 | 0.03 | 1.21 | -1.42 | 0.66 |
|  | 95%CI | [-0.44,0.81] | [-0.47,0.86] | | | | [-0.53,0.79] | | | | [-0.67,0.61] | | | [-1.03,0.39] | [-0.62,0.71] | [0.38,2.05] | [-2.33,-0.52] | [-0.02,1.43] |

Note: Age and Musical training are in years; NVIQ and ROWPVT-IV are percentile points of nonverbal IQ and standard scores of receptive verbal ability respectively; Corsi and Digit span are the raw scores of nonverbal and verbal short-term memory respectively; AQ, EQ and SQ are the scores of Autism Spectrum, Empathy and Systemizing Quotient respectively. Bayes factors from a default prior 2-tailed Bayesian Mann-Whitney-Wilcoxon Test are expressed in terms of the Bayes factor in favour of the null hypothesis of no difference (BF_01_). The delta effect size in these Bayesian comparisons is given by the median of a posterior distribution and 95% credible intervals.
